# Evaluation of oral mucositis severity and management in head and neck cancer patients undergoing radiotherapy: A prospective study

**DOI:** 10.6026/973206300212104

**Published:** 2025-07-31

**Authors:** Paijas K.M., Punit G. Naidu, Onteru Pradeep, Heena Dixit, Deepak Sharma, Rahul Tiwari, Anil Managutti

**Affiliations:** 1 Department of Oral Medicine and Radiology, Al Azhar Dental College, Thodupuzha, Kerala, India; 2 Department of Periodontology, Index Institute of Dental Sciences, Indore, Madhya Pradesh, India; 3 Department of Public Health Dentistry, Father Colombo Institute of Medical Sciences, Warangal, Telangana, India; 4 Department of Hospital Administration, Index Institute, Malwanchal University, Index City, Nemawar Road, Indore, Madhya Pradesh, India; 5 Department of Oral and Maxillofacial Surgery, Narsinhbhai Patel Dental College and Hospital, Sankalchand Patel University, Visnagar, Gujarat, India

**Keywords:** Head and neck cancer, oral mucositis, radiotherapy, chemoradiotherapy, mucosal toxicity

## Abstract

Oral mucositis is a frequent and debilitating side effect in patients undergoing radiotherapy for head and neck cancers, often leading to pain, nutritional compromise and treatment interruptions. A prospective observational research was conducted on 60 patients undergoing radiotherapy, with or without chemotherapy. 46.7% of patients developed Grade 3-4 mucositis. Severe mucositis was significantly associated with low BMI (p = 0.031), hemoglobin <11 g/dL (p = 0.007), tumor site (p = 0.016) and concurrent chemoradiotherapy (p = 0.001). Mucositis severity is influenced by nutritional and treatment-related factors. Early identification and targeted supportive interventions can improve clinical outcomes and reduce treatment disruptions.

## Background:

Oral mucositis (OM) is a frequent and debilitating side effect experienced by patients undergoing radiotherapy (RT) for head and neck cancers [1]. Associated by erythema, ulceration and pain in the oral mucosa, mucositis significantly compromises patients' nutritional intake, quality of life and adherence to cancer treatment protocols [2]. The prevalence of OM in head and neck cancer patients undergoing RT approaches 80-100%, with varying degrees of severity depending on individual susceptibility, RT dose fractionation and concurrent therapies like chemotherapy or targeted agents [3]. The biological mechanism underlying OM involves a complex, five-phase cascade: initiation, up regulation with messenger generation, signaling and amplification, ulceration and healing [4]. During RT, mucosal cells undergo DNA damage and generate reactive oxygen species, leading to activation of nuclear factor-kappa B and subsequent release of pro-inflammatory cytokines [4]. These molecular events culminate in mucosal breakdown, ulcer formation and heightened risk of local and systemic infections [5]. Assessment of OM severity is typically performed using validate scoring systems like the "World Health Organization (WHO") Oral Toxicity Scale or the "National Cancer Institute Common Terminology Criteria for Adverse Events (NCI-CTCAE)". These tools are crucial not only for clinical decision-making but also for evaluating the efficacy of various prophylactic and therapeutic interventions [6]. Recent advancements have focused on cytoprotective agents, low-level laser therapy and natural antioxidants; however, the comparative effectiveness of these modalities remains under investigation. The burden of OM is not limited to physical symptoms. Severe grades of mucositis often result in unplanned treatment interruptions, increased hospitalization duration and a higher economic burden on healthcare systems [7]. Moreover, mucositis-induced pain necessitates opioid use in a substantial proportion of cases, thereby compounding complications related to sedation, constipation and dependency [8]. Given this clinical impact, early prediction and management of OM severity are vital components of multidisciplinary cancer care. Recent clinical studies have explored several patient- and treatment-related risk factors that may influence the development of OM, including baseline nutritional status, oral hygiene, age, gender, tumor site and RT dosimetric parameters [9]. Yet, a predictive model based on prospective clinical data integrating these parameters remains largely underdeveloped, especially in low- and middle-income settings. Moreover, while several interventions have been individually tested, robust comparative data on treatment outcomes using multimodal supportive care protocols are limited [10]. Therefore, it is of interest to not only validate predictive associations but also evaluate the effectiveness of commonly adopted mucositis management protocols in a real-world, resource-constrained clinical setting.

## Materials and Methods:

## Research design and setting:

A prospective, observational research conducted in the Radiation Oncology Department of a tertiary care hospital over a period of 18 months. Consents and ethical approvals were obtained for current research.

## Subject selection:

Inclusion criteria were adult patients (≥18 years) diagnosed with histologically confirmed head and neck squamous cell carcinoma, planned for external beam radiotherapy (with or without concurrent chemotherapy) and with no prior history of radiotherapy. Exclusion criteria included pre-existing oral mucosal lesions, known autoimmune mucosal disorders, uncontrolled diabetes and poor baseline oral hygiene unamenable to standard intervention.

## Treatment protocol:

All patients received conventionally fractionated radiotherapy (2 Gy/day, 5 days/week) up to a total dose of 60-70 Gy, using either 3D conformal RT or IMRT based on tumor location. Concurrent chemotherapy, when administered, consisted of cisplatin 40 mg/m^2^ weekly. All patients received baseline dental evaluation and prophylactic oral care prior to initiation of therapy.

## Assessment of OM:

Patients were assessed twice weekly for OM throughout the radiotherapy course and up to 2 weeks post-treatment. The WHO Oral Toxicity Grading Scale was used to categorize OM severity (Grade 0 to 4). Data on onset, peak severity and resolution time were recorded.

## Management interventions:

Supportive care included patient-specific interventions such as saline rinses, topical anesthetics, oral antifungals, multivitamins and analgesics. In moderate to severe cases (Grades 3-4), topical corticosteroids and systemic analgesics including opioids were prescribed. Selected patients also received low-level laser therapy (LLLT) based on resource availability. Nutritional support was provided through high-protein supplements or nasogastric feeding where required.

## Data collection and statistical analysis:

Demographic variables (age, gender, BMI), clinical factors (tumor site, stage, treatment modality) and baseline laboratory markers (hemoglobin, serum albumin) were recorded. Outcomes included OM grade, time to onset, duration and need for treatment modification. All data were entered into Microsoft Excel and analyzed using SPSS version 26.0. Descriptive statistics (mean ± SD, frequencies and percentages) were used for baseline characteristics. Chi-square test or Fisher's exact test was used for categorical variables. A p-value of <0.05 was considered statistically significant.

## Results:

Of the 60 patients enrolled, 28 (46.7%) developed severe mucositis (Grade 3-4), while 32 (53.3%) had Grade 0-2. Severe mucositis was significantly associated with low BMI (<18.5 kg/m^2^, p = 0.031), low hemoglobin (<11 g/dL, p = 0.007), and concurrent chemoradiotherapy (p = 0.001). Tumors in the oral cavity and oropharynx showed higher mucositis rates than laryngeal sites (p = 0.016). These findings highlight the influence of nutritional, hematologic, and treatment factors on mucositis severity (Table 1 - see PDF). Table 2 (see PDF) summarizes the clinical outcomes and interventions based on mucositis severity. Patients with Grade 3-4 mucositis had significantly longer recovery times (mean 16.2 days vs. 9.3 days, p < 0.001), higher need for opioid analgesics (71.4% vs. 28.1%, p = 0.001) and required more frequent nutritional interventions (p = 0.012). Treatment interruptions (≥3 days) were noted in 25% of severe cases, while none were reported in milder cases (p = 0.008). Use of low-level laser therapy (LLLT) showed a trend toward faster mucosal recovery but did not reach statistical significance (Table 2 - see PDF).

## Discussion:

The present research evaluated the severity and outcomes of OM in head and neck cancer patients undergoing radiotherapy, identifying key predictive factors and assessing clinical management responses. Nearly half of the research participants developed severe mucositis (Grade 3-4), consistent with previous reports highlighting mucositis as a common complication of head and neck radiation therapy [11]. Significant associations were observed between lower BMI, anemia and concurrent chemoradiotherapy with higher mucositis severity. Malnourished patients exhibited impaired mucosal healing, potentially due to deficiencies in protein synthesis and immune function. Hypoalbuminemia, although not statistically significant, trended toward greater mucosal breakdown, aligning with earlier evidence linking nutritional status to mucositis risk [12]. Similarly, anemia appeared to be a reliable predictor of increased mucosal toxicity, possibly due to compromised oxygen delivery to regenerating epithelial tissues. Tumor site emerged as a determinant of severity, with oral cavity and oropharyngeal tumors demonstrating higher mucositis incidence. This may be attributed to their proximity to high-dose radiation fields, which exposes a larger surface area of the oral mucosa to cumulative cytotoxic effects [13]. Additionally, patients receiving concurrent chemoradiotherapy had significantly higher mucositis grades, reaffirming the additive toxicity of systemic agents on mucosal integrity. Management outcomes revealed that severe mucositis correlated with prolonged recovery times, increased opioid use and greater dependency on nutritional support. These findings highlight the multidimensional burden of mucositis, not only in terms of pain and feeding difficulty but also due to potential interruptions in cancer treatment, which were observed in 25% of severe cases [14, 15]. The present study's findings showing a progressive increase in mucositis severity and associated QoL impairment align with those reported by Franco *et al.*, who used validated assessment tools (OMAS, OMWQ-HN, and FACT-HN) to document the temporal evolution of oral mucositis and its significant negative impact on patient-reported outcomes during head and neck radiotherapy [16]. Although low-level laser therapy showed some promise in hastening recovery, statistical significance was not achieved, likely due to sample size limitations. The results support the need for early identification of high-risk patients and timely implementation of preventive strategies. Predictive models integrating clinical and nutritional parameters may be valuable tools for individualized risk stratification. Furthermore, adopting multimodal management approaches-including pain control, nutritional counseling and mucosal protectants-may improve patient outcomes and minimize treatment disruptions [15, 16, 17, 18, 19-20].

## Conclusion:

This prospective study demonstrates that OM remains a significant adverse effect of radiotherapy in head and neck cancer patients. Severity is influenced by nutritional status, hemoglobin levels, tumor location and concurrent chemo-radiation. Timely intervention and supportive care can reduce complications, opioid use and treatment delays. Future clinical practice should focus on early risk identification and evidence-based management strategies to improve quality of life and treatment adherence in this patient population.
